# Efficient tuning of zinc phthalocyanine-based dyes for dye-sensitized solar cells: a detailed DFT study†

**DOI:** 10.1039/d1ra04529f

**Published:** 2021-08-12

**Authors:** Sabir Ali Siddique, Muhammad Arshad, Sabiha Naveed, Muhammad Yasir Mehboob, Muhammad Adnan, Riaz Hussain, Babar Ali, Muhammad Bilal Ahmed Siddique, Xin Liu

**Affiliations:** Center for Organic Chemistry, School of Chemistry, University of the Punjab Lahore-54590 Pakistan; Institute of Chemistry, The Islamia University of Bahawalpur, Baghdad-ul-Jadeed Campus Bahawalpur-63100 Pakistan muhammad.arshad@iub.edu.pk; Department of Chemistry, University of Okara Okara-56300 Pakistan riazhussainansari@gmail.com; Graduate School, Department of Chemistry, Chosun University 501-759 Gwangju Republic of Korea; Department of Physics, University of Okara Okara-56300 Pakistan; School of Chemistry and Chemical Engineering, Shandong University Jinan-250100 China bilal.siddique@mail.sdu.edu.cn; State Key Laboratory of Fine Chemicals, Department of Chemistry, Dalian University of Technology Dalian 116024 P. R. China xliu@dlut.edu.cn

## Abstract

The growing energy demand speed up the designing of competent photovoltaic materials. Herein, five zinc phthalocyanine-based donor materials T1–T5 are designed by substituting various groups (isopropoxy, cyano, fluoro, methoxycarbonyl, and dicyanomethyl) around zinc phthalocyanine. B3LYP/6-31G (d,p) level density functional theory (DFT) was used to investigate the optoelectronic properties of five zinc phthalocyanine-based dyes T1–T5 for dye-sensitized solar cells. The designed molecule T1 shows maximum absorption wavelength (*λ*_max_) in the absorption spectrum at 708.89 and 751.88 nm both in gaseous state and in THF (tetrahydrofuran) solvent. The *E*_g_ value of T1 (1.86 eV) is less than reference R, indicating a greater charge transfer rate for T1 among the molecules. The values of open-circuit voltages achieved with acceptor polymer PC_71_BM are higher than R except for T1 and are 0.69 V, 1.95 V, 1.20 V, 1.44 V, and 1.84 V for T1, T2, T3, T4, and T5, respectively. The lower the reorganization energy, the higher the charge transfer for T1 due to its lower hole mobility (0.06297 eV) than R. Thus, the designed T1–T5 molecules are expected to exhibit superior performance in dye-sensitized solar cells.

## Abstract 2

We used a quantum chemical approach to investigate the optoelectronic properties of dyes T1–T5 for dye-sensitized solar cells using DFT and TD-DFT computation. The newly designed molecules exhibited outstanding photovoltaic and optoelectronic properties.

## Graphical abstract

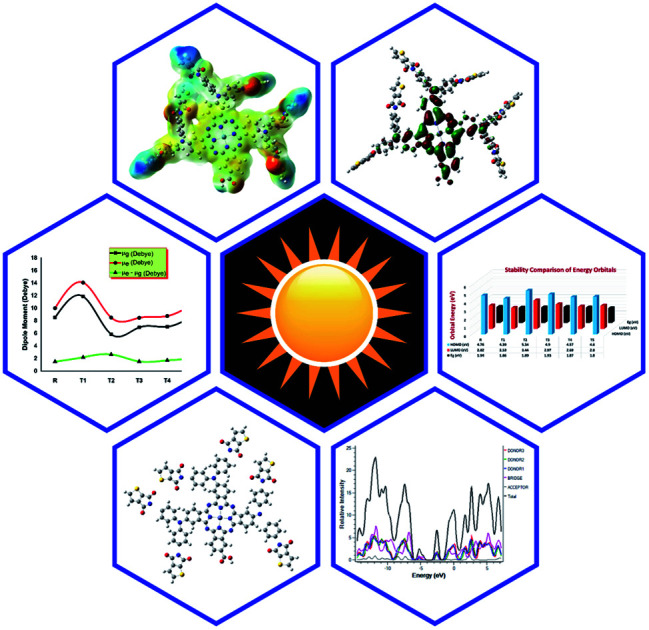

## Introduction

Dye-sensitized solar cells (DSSCs) have originated as an economically credible photovoltaic technology and are considered a promising alternative to conventional p–n junction devices.^1^ Since they have already demonstrated their worth for the society by providing more than 13% power conversion efficiency (PCE) at the laboratory scale, while around 10% in small photovoltaic modules.^2^ Due to the remarkable achievements under various irradiation conditions, DSSCs are considered an ideal candidate under artificial light, shadowed or dim environments.^3^ Their manufacturing process is quite simple, cost-effective, environmentally friendly, and compatible to meet the industrial requirements to produce large-area devices.^4^

However, during the preparation of transparent SCs, there is a need for a compromise between the efficiency and transparency of devices. Therefore, a lot of efforts have been made to overcome the issue and prepare state-of-the-art devices beyond the limit of fixing optical transmissions while fabricating semi-transparent DSSCs devices. Primarily, the ruthenium-based DSSCs offered good optical and photovoltaic characteristics and are quite prominent as compared with previously used DSSCs, but their usage was limited due to very small absorption in the near-infrared region with lower intensities.^5^ Therefore, due to this, another family of dyes, such as phthalocyanines, offered good and efficient absorption properties when used in DSSCs because of their integrated structure and charge shifting behavior.^6^

In recent years, the use of phthalocyanines is quite attractive due to their specific merits such as they are electrochemically, photo-chemically, thermally stable species, and this is because of their higher absorption capability in the near and far-infrared region.^7^ Importantly, absorption in the near-infrared region with maximum intensity is associated with phthalocyanines when used in DSSCs. The light absorption bands in phthalocyanines are shifted towards the red/infrared region of radiations due to the positioning of peripheral and non-peripheral functional groups on the macrocycle.

In phthalocyanines, Zn-phthalocyanines are the successful candidates for DSSCs because of electron-donating units, which are the key constituents on the macrocycle. Three electron donor units are on the isoindole sub-unit (named as A) of the macrocycle, while the fourth unit is present on the anchoring group that directly interacts with metal oxide.^8^ This A3B type configuration of Zn-phthalocyanines allows less steric hindrance with efficient push–pull effects making Zn-phthalocyanines an efficient absorber of sunlight in the near-infrared region.^9,10^

In the literature, studies disclosed the presence of substituents on A and B units, like electron-rich unit (2,6-bis(*n*-butoxy)phenoxy) group on A and electron catching group such as COOH on B that directly boosted PCEs up to 6.4%, which is more than reported previously by Nazeeruddin.^11^ Besides these reports, research is going on in this field with continuous increments in PCE values. Recently, Milan and his co-authors efficiently designed and demonstrated Zn-phthalocyanines for DSSCs.^12^ They studied peripherally substituted push–pull Zn-phthalocyanines bearing three electron-donating diphenylamine substituents and a carboxylic acid anchoring group and integrated it as a sensitizer in TiO_2_-based DSSCs. They concluded that the synthesized molecule offered great absorption in the near-infrared region, which directly enhanced the light-harvesting efficiencies.

In DSSC, sensitizers with extended absorption in the near IR region of the solar emission spectrum are essential, and phthalocyanines are excellently suitable for their integration in light energy conversion systems. Therefore, asymmetric Zn-phthalocyanine is being adapted as a standard material showing exceptional light to energy efficiency in DSSCs. The asymmetric phthalocyanines with higher light-harvesting effects result in higher photocurrents and their potential use.^13–15^ The recent studies highlight tetra triphenylamine-substituted Zn-phthalocyanine as a hole transporting material and highlight the importance of a buffer layer between the perovskite layer and the hole-transporting layer.^16^

Herein, we designed a new strategy to further improve the absorption/light-harvesting capabilities of Zn-phthalocyanines by incorporation of different end-capped units. With these modifications, we efficiently designed five new molecules that offer high absorption in the near-infrared region with maximum intensity. After designing, we theoretically characterized these materials by computing geometric parameters and physicochemical properties. Moreover, the density of states analysis, alignment of frontier molecular orbitals, reorganizational energy of holes and electrons, transition density matrix, and photovoltaic properties of these newly designed materials are investigated. The outcome from all theoretical parameters suggested that these designed molecules are good candidates for high-performance DSSCs applications.

## Computational details

All the computations were carried out on designed molecules as well as the reference molecule using the Gaussian 09 package.^17^ The structures of molecules were drawn with the help of Gauss View 6.0.^18^ Initially, the absorption spectra and geometry of reference compound R^19^ were optimized using CAM-B3LYP, B3LYP, WB97XD MPW1PW91, HSEH1PBE, PBEPBE, and TPSSTPSS functionals with 6-31G (d,p) basis set and the results compared with experimental data. B3LYP at 6-31G (d,p) basis set provides the strongest agreement between experimental and theoretical results. Thus, this method was selected for further calculations. Both the electronic and optical properties like the density of states, maximum absorption (*λ*_max_), frontier molecular orbitals (FMOs), transition density matrix (TDMs), and reorganization energy are calculated using the B3LYP/6-31G (d,p) method of DFT.

Open circuit voltages (*V*_oc_) explain the amount of current that can be passed through any material. The difference between HOMO_donor_ and LUMO_acceptor_ is known as open-circuit voltage. Reorganization of energy is the main point of charge mobilities. There are two parts of reorganization energy, internal and external. Relaxation in the environment is studied with the help of external reorganization (*λ*_h_), and change in molecular geometry is studied with internal reorganization (*λ*_e_). In this study, we only deal with internal reorganization (*λ*_e_).^20^ The equations of reorganization energies are as following:

1 *λ*_e_ = [*E*^−^_0_ − *E*_−_] + [*E*^0^_−_ − *E*_0_]

2 *λ*_h_ = [*E*^+^_0_ − *E*_+_] + [*E*^0^_+_ − *E*_0_]

In the above equations, *E*^0^_−_ and *E*^0^_+_ are the energies of neutral molecules at the anionic and cationic states, respectively. *E*_+_, *E*_−_ indicates the energies of cation and anion, respectively, *via* optimized geometries of cation and anion molecules. *E*^+^_0_ and *E*^−^_0_ are the energies of cation and anion with the optimized structure of neutral molecules. Finally, *E*_0_ is the energy of neutral molecules at the ground state.

## Results and discussion

**Fig. 1:**
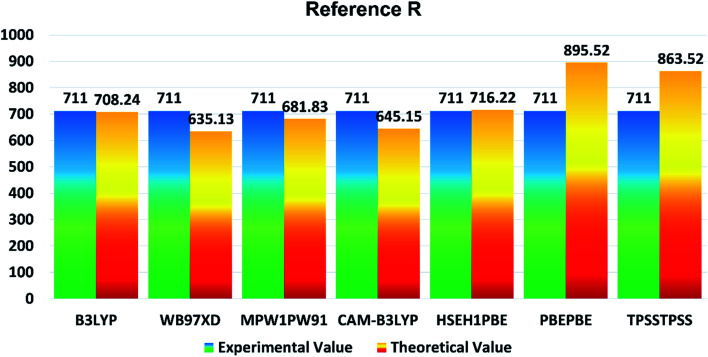
Comparison diagram of *λ*_max_ of R between experimental and theoretical values with B3LYP, MPW1PW91, CAM-B3LYP, ωB97XD, HSEH1PBE, PBEPBE and TPSSTPSS at 6-31G (d,p).

In the present study, B3LYP, ωB97XD, MPW1PW91, CAM-B3LYP, HSEH1PBE, PBEPBE, and TPSSTPSS functionalities with 6-31G (d,p) basis set were applied for initial optimization of the reference molecule R to determine the optoelectronic properties. The maximum absorption (*λ*_max_) values of reference R with B3LYP, ωB97XD, MPW1PW91, CAM-B3LYP, HSEH1PBE, PBEPBE, and TPSSTPSS are found to be: 708.24 nm, 635.13 nm, 681.83 nm, 645.15 nm, 716.22 nm, 895.52 nm, and 863.52 nm, respectively. The *λ*_max_ of reference R obtained experimentally is 711 nm ^19^ and shown in Fig. 1. The B3LYP/6-31G (d,p) method of DFT has the strongest relationship with the experimental *λ*_max_ value and it is therefore ideal for further calculations of designed molecules T1–T5.

**Fig. 2:**
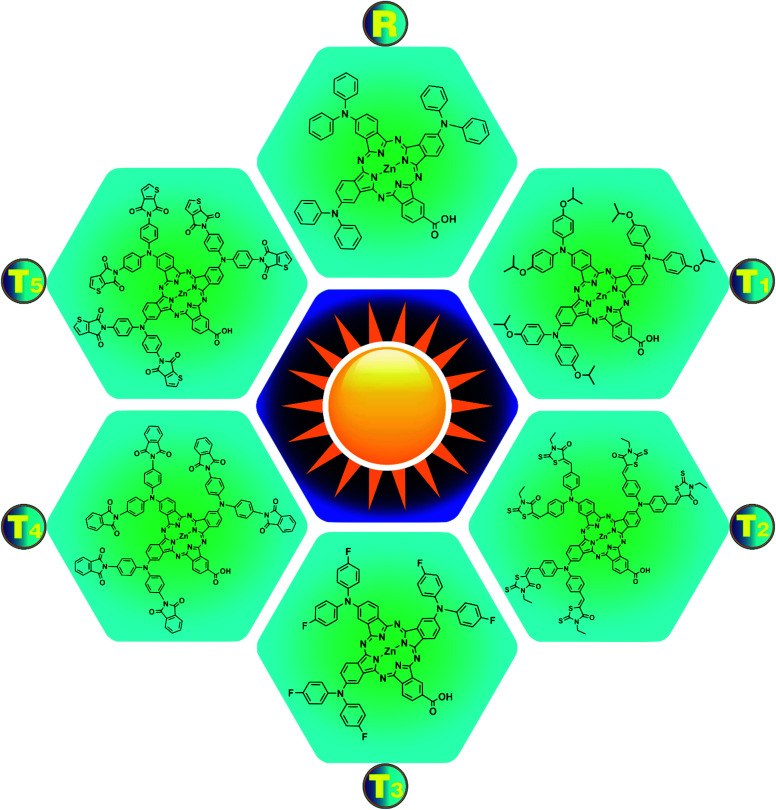
Structural formulas of reference compound R and designed molecules T1–T5.

This report aims to improve the photovoltaic properties of zinc phthalocyanine-based donors *via* modification of donor moieties. For this, a newly synthesized molecule, (23-carboxy-2,9,16-tris(diphenylamino)phthalocyanine-29-yl)zinc(ii) complex R^19^ is modified using specific donor groups like (isopropoxy, cyano, fluoro, methoxycarbonyl, and dicyanomethyl). The modified structures of all newly designed molecules T1–T5 and reference R are expressed in Fig. 2. Effective molecular design is the key to boosting photovoltaic efficiency by decreasing the energy gap (*E*_g_) by operating charge transfer (CT) within the molecule and regulating its energy levels. The optimization of the ground state and the planar conformation of optimized structures of the reference R and designed molecules T1–T5 are shown in Fig. S1,† and it shows that the planar conformations promote the mobility of charges in molecules (T1, T2, T3, T4, and T5) due to increased conjugation present in these systems by the delocalization of electrons.

**Fig. 3:**
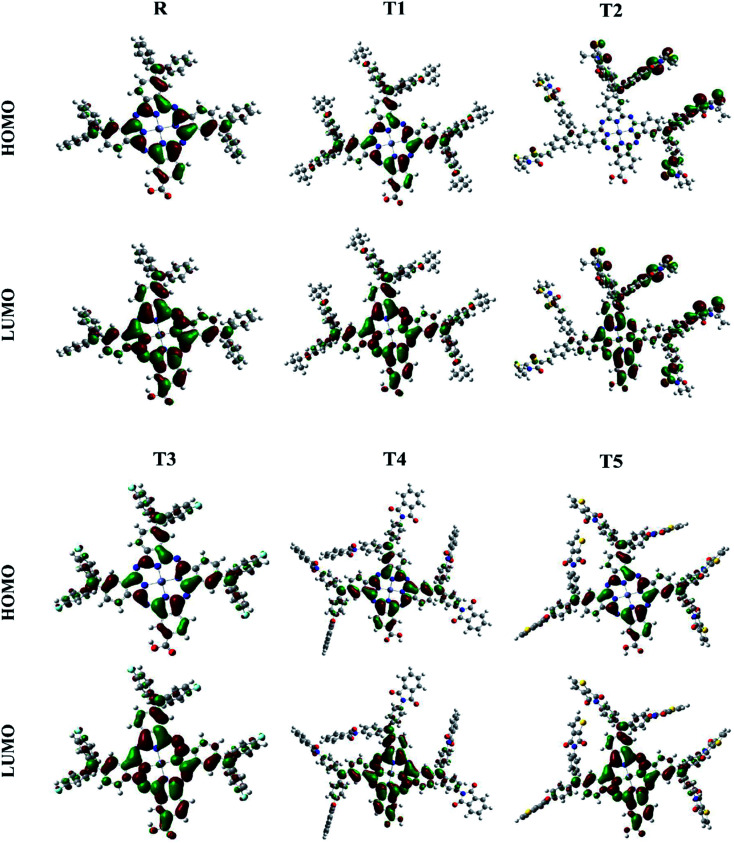
Frontier molecular orbital diagrams of reference R and compounds T1–T5 at B3LYP/6-31G (d,p) level of theory.

The photovoltaic efficiency of organic solar cells can be improved by a small energy gap (*E*_g_). The HOMO and LUMO of molecular orbitals clarify the bonding and anti-bonding character of compounds.^21^ The amount of current, voltage, and effectiveness of the cell is directly proportional to the transfer of electrons from the donor part to the acceptor part of the molecule. The transfer of electrons depends on the method of electron excitation. Thus, the transfer of an electron from the donor to the acceptor can be improved by improving the method of electron excitation.^22^ In Fig. 3, frontier molecular orbitals of compounds are represented as a distribution around HOMO and LUMO. The reference R and T1–T5 molecules were optimized with B3LYP at the 6-31G (d,p) basis set level. The calculated band gap (*E*_g_) values between HOMO and LUMO are set as a stability index and correspond to the difference between HOMO–LUMO energies.^23^

**Fig. 4:**
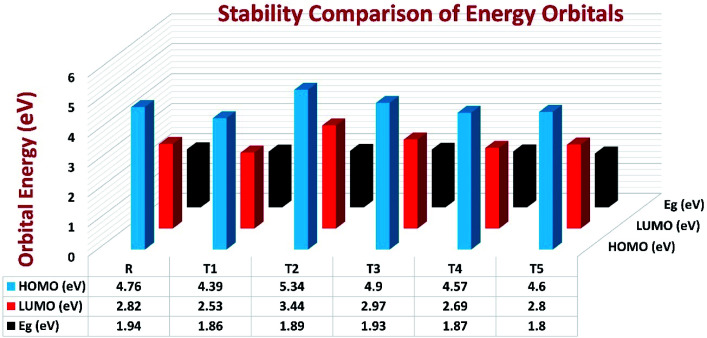
Orbital energies of designed donor molecules T1–T5 and reference molecule R at B3LYP/6-31G (d,p) level of theory.

To avoid self-aggregation, the donor and acceptor parts of the newly designed compounds were set in the 3D method. The electronic and optical characteristics of molecules T1–T5 were characterized by frontier molecular orbital theory distribution. The *E*_HOMO_ and *E*_LUMO_ of the molecule R were −4.76 and −2.82 eV, respectively, and the band gap was 1.94 eV, as shown in Table 1. The *E*_HOMOs_ and *E*_LUMOs_ of new compounds T1, T2, T3, T4, T5 were −4.39, −5.34, −4.90, −4.57, −4.60 eV, and −2.53, −3.45, −2.97, −2.69, −2.80 eV, respectively, as described in Fig. 4. Besides the significance of HOMO and LUMO energies, their band gaps are also important. The band gap value of compound R was 1.94 eV, while T5 has the lowest (1.80 eV) band gap than all other molecules due to the isopropoxy group bearing the donor characteristics.

**Table 1:** Energies of HOMOs and LUMOs and HOMO–LUMO gap at B3LYP/6-31G (d,p) level of theory for reference compound R and designed molecules T1, T2, T3, T4, and T5

Molecules	*E*_HOMO_ (eV)	*E*_LUMO_ (eV)	*E*_g_ (eV)
R	−4.76	−2.82	1.94
T1	−4.39	−2.53	1.86
T2	−5.34	−3.45	1.89
T3	−4.90	−2.97	1.93
T4	−4.57	−2.69	1.87
T5	−4.60	−2.80	1.80

The *E*_g_ values of the five newly designed compounds are 1.86, 1.89, 1.93, 1.87, 1.80 eV, respectively. The T5 molecule has the lowest band gap than reference molecule R, which means this molecule can display an easy charge transformation method. Comparatively low HOMO values of designed compounds will accelerate the open-circuit voltage of dye-sensitized solar cells.

### Density of state (DOS) and overlap population density of state (OPDOS)

To obtain details on the increase in energy levels per unit, OPDOS and DOS studies of all the newly planned compounds were performed, and their results support the findings obtained from the frontier molecular orbitals given in Fig. 3. The higher value of DOS is that there are a significant number of states for these energy levels. The zero values of OPDOS and DOS indicate that there is no state available to occupy the electrons for this level of energy.^24,25^

**Fig. 5:**
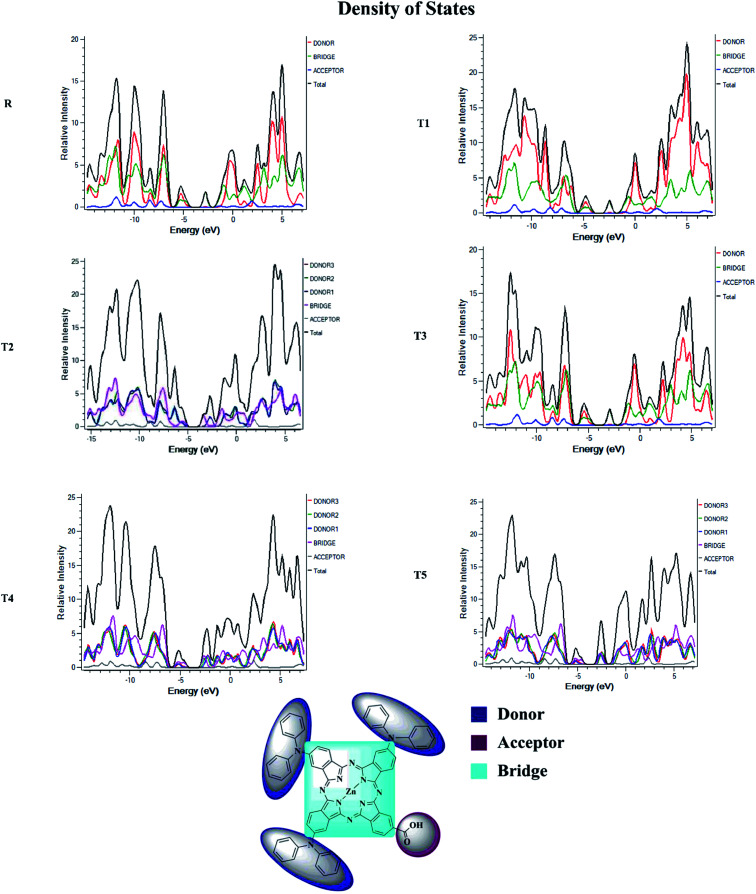
DOS map for reference R and molecules T1–T5 at B3LYP/6-31G (d,p) level of theory.

**Fig. 6:**
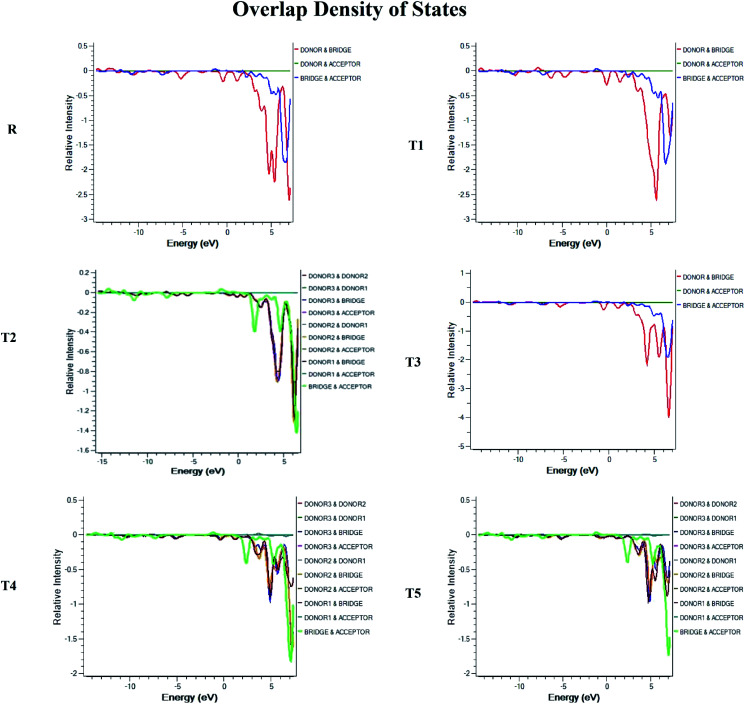
OPDOS map for reference R and molecules T1–T5 at B3LYP/6-31G (d,p) level of theory.

Generally, DOS represents the space occupied by the system. DOS and OPDOS for molecules T1–T5 and reference R at B3LYP/6-31G (d,p) method of DFT are given in Fig. 5 and 6, respectively. OPDOS and DOS studies help clarify the contribution of donor–acceptor species in the formation of frontier molecular orbitals. DOS study illustrates the energy of occupied and unoccupied MO's of designed molecules. In the spectrum, green and blue colors represent the energy states to occupy for the electron around the donor part of the compound, while red color defines the acceptor moiety.

In the graph of density of state, the space between the acceptor and donor part of the compound is represented by the band gap (*E*_g_). The band gap is the energy needed for electron excitation.^26^ In this study, smaller energy is required to excite the electrons from the acceptor to the donor part for all the compounds.

### Optical properties

The optical characteristics of photovoltaic compounds (T1–T5) were predicted with the TD-B3LYP/6-31G (d,p) method of DFT and are given in Table 2. The design feature of effective photovoltaic compounds is based on their strong and comprehensive absorption properties. Firstly, to observe the photophysical properties in both the gaseous and THF solvent phases, molecule R was optimized with different functionals like TD-ωB97XD, TD-B3LYP, TD-CAMB3LYP, TD-MPW1PW91, TD-HSEH1PBE, TD-PBEPBE, and TD-TPSSTPSS at 6-31G (d,p) method of DFT.

**Table 2:** Calculated wavelength (*λ*_max_), experimental wavelength (*λ*_max_), excitation energies (*E*_x_) and oscillator strength (*f*), dipole moment (*μ*), % electron transport contributions (%ETC) of reference R and designed molecules T1, T2, T3, T4, and T5 at B3LYP/6-31G (d,p) level of theory in the gas and solvent phases, respectively

	Molecules	Calc. *λ*_max_ (nm)	Exp. *λ*_max_ (nm)	*f* (a.u)	Assignment	*E*_x_ (eV)	Dipole moment (debye)
Gaseous	R	680.11	711	0.64	HOMO → L+1 (93%)	1.823	8.511
	T1	708.89	—	0.69	HOMO → L+1 (94%)	1.749	11.875
	T2	708.93	—	0.71	HOMO → LUMO (63%), HOMO → L+1 (30%)	1.749	5.861
	T3	678.51	—	0.65	HOMO → L+1 (93%)	1.827	6.942
	T4	671.16	—	0.76	HOMO → L+1 (91%)	1.847	7.054
	T5	682.88	—	0.42	HOMO → L+1 (86%)	1.816	9.056
Solvent	R	708.24	711	0.81	HOMO → L+1 (96%)	1.751	10.007
	T1	751.88	—	0.82	HOMO → L+1 (96%)	1.649	14.073
	T2	706.46	—	0.97	HOMO → LUMO(58%), HOMO → L+1 (37%)	1.755	8.515
	T3	706.38	—	0.81	HOMO → L+1 (96%)	1.755	8.469
	T4	697.95	—	0.94	HOMO → L+1 (95%)	1.776	8.763
	T5	695.17	—	0.87	HOMO → L+1 (95%)	1.783	10.919

**Fig. 7:**
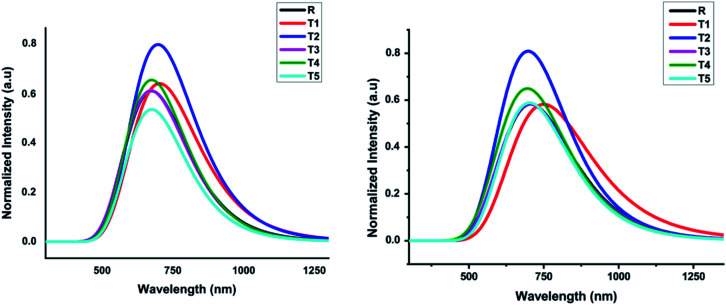
Calculated absorption spectrum of all designed molecules (T1–T5) and reference R at TD-B3LYP/6-31G (d,p) in gas and solvent (THF) phases, respectively.

In comparison, the results obtained from the TD-B3LYP/6-31G (d,p) DFT method were consistent with the experimental UV spectra of compound R. The theoretical study exposed that the absorption spectrum (*λ*_max_) of the reference compound in THF solvent was 708.24 nm and 680.11 nm in the gaseous phase. The experimental absorption value of the reference compound was 711 nm that is similar to the value obtained in THF solvent. Therefore, we used the TD-B3LYP/6-31G (d,p) method to compute the photophysical characteristics of all the newly designed compounds T1–T5. For all these compounds and reference R, 10 ETS (electronic transition excited states) were generated in THF solvent as well as in the gaseous phase. The experimental wavelength (*λ*_max_), calculated wavelength (*λ*_max_), oscillator strength (*f*), %ETC (electron transport contribution), dipole moment, and excitation energy (*E*_x_) for all compounds T1–T5 at TD-B3LYP/6-31G (d,p) method of DFT in the gas and solvent (THF) phases are shown in Table 2. The calculated *λ*_max_ in the gas phase for designed compounds T1–T5 and reference R at TD B3LYP/6-31G (d,p) was in the range of 652.34–708.89 nm, as shown in Fig. 7.

The compounds T1–T5 and reference R display strong and broad spectral peaks, ranging from 600 nm to 1000 nm. Both the designed and reference molecules display one large peak in the range from 600 to 900 nm. The molecule T1 has shown higher *λ*_max_ than reference R in the gaseous phase. With an oscillator strength of 0.64, the *λ*_max_ of reference R was 680.11 nm in the gaseous phase. For the T1 molecule, the absorption band is red-shifted than T2, T3, T4, and T5 designed molecules with increased oscillator strength (*f*) up to 0.70. The obtained oscillator strengths (*f*) of T1, T2, T3, T4, and T5 molecules were 0.69, 0.71, 0.65, 0.76, and 0.42, respectively.

The compound T5 is blue-shifted in the ultraviolet-visible spectrum at the lowest oscillator strength 0.42 with *λ*_max_ 682.88 nm than compound R. The electron transport contributions percentage (%ETC) in both phases, gaseous and solvent, changes from 81% to 94% and 93% to 96%, respectively. If the %ETC is smaller, excitation chances will be greater for T1–T5 designed molecules. Among all newly designed molecules, T2 and T5 have lower %ETC values both in gaseous and solvent phases, lower than the reference compound in both phases. The chances for the excitation of electrons of T2 and T5 are greater due to lower %ETC.

### Molecular electrostatic potential

Molecular ESP calculations were performed to compare rich and deficient sites of electrons in T1–T5 designed molecules with reference R. The properties of the compounds were represented by three colors (green, red, blue) in the MEP graphs. The green color shows the neutral, the blue color indicates the positive charge accumulation (electron-efficient) part, and red represents the negatively charged accumulation (electron-deficient) part of the compound.^27^

**Fig. 8:**
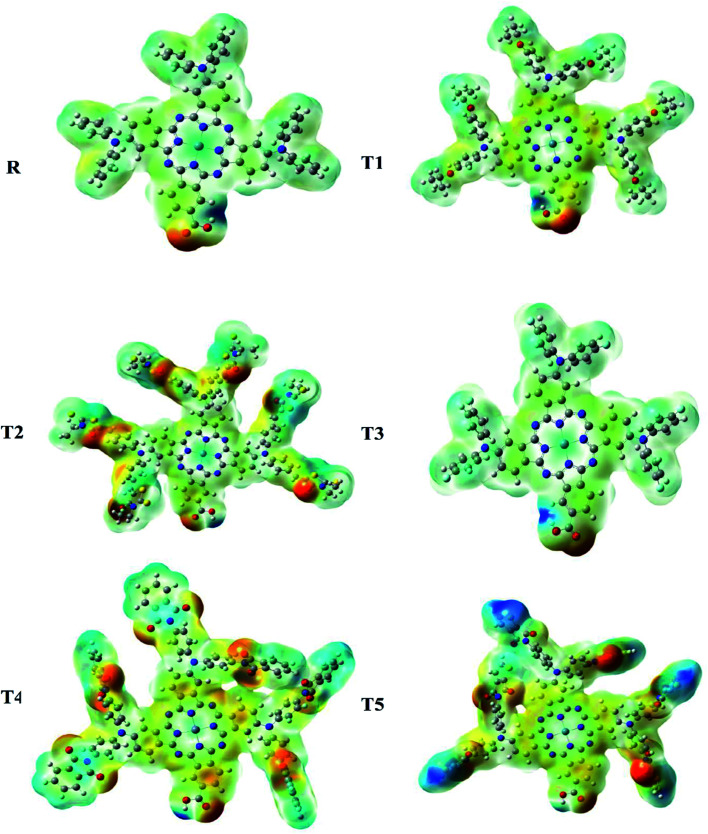
Molecular electrostatic potential analysis of designed (T1–T5) and reference R molecules.

Interestingly, it was observed that the distribution pattern of all compounds, as well as reference, was the same that indicates all the designed compounds can be used for solar cells. Therefore, all newly designed molecules are the best candidates for solar cell applications due to similar MEP graphic distribution to the R reference molecule (Fig. 8).

### Reorganization energy

Dye-sensitized solar cell performance relies on the charge mobilities. The reorganization energies of electron (*λ*_e_) and hole (*λ*_h_) mobilities help to calculate the charge mobilities. The evaluation of the relation between the transportation of charges and the structure of molecules is important for the improvement of photovoltaic materials. The reorganization energy has an inverse relationship with the mobilities of charges. As the value of reorganization energy is higher, lower will be the mobility of charges of the newly designed compounds.

For the analysis of reorganization energies of reference R and compounds T1–T5, the geometries of the anions and cations were used. The values for reorganization energy of all the molecules as well as reference are given in Table 3. This analysis suggests that the designed compound T4 has better *λ*_e_ than the reference and other designed compounds due to isoindoline-1,3-dione substituent. The reorganization energy value of *λ*_h_ as compared to *λ*_e_ indicates T1 is good for the mobility of charges. The *λ*h value of the reference is greater than the designed donor compound T1, which means that the mobility of charges of the reference is lower than the T1 compound. The values for electron mobilities *λ*_e_ for reference as well as other compounds T1–T5 are 0.19217, 0.19631, 0.14933, 0.19986, 0.07197, and 0.20844 eV, correspondingly.

**Table 3:** Reorganization energies of reference R and designed molecules T1–T5

Molecules	*λ*_e_ a (eV)	*λ*_h_ b (eV)
R	0.19217	0.08712
T1	0.19631	0.06297
T2	0.14933	0.07883
T3	0.19986	0.08122
T4	0.07197	0.10100
T5	0.20844	0.12448

a Reorganization energy of the electron.

b Reorganization energy of the hole.

The newly designed compound T2 has a lower value of *λ*_e_ than reference, which indicates this compound has greater mobility of charges than the reference compound. The mobility of holes *λ*_h_ for reference as well as other molecules T1–T5 is 0.08712, 0.06297, 0.07883, 0.08122, 0.10100, 0.12448 eV, respectively. The T1 molecule has a low value of *λ*_h_ than reference and other designed molecules. The least value of reorganizational energy of holes in the T1 molecule suggested that the T1 molecule is a potential candidate for efficient hole transport material. The analysis of reorganization energies proves that T1 is the best for hole transport mobility.

### Dipole moment

The solubility of dye-sensitized solar cell materials is mainly dipole moment dependent. Thus, the dipole moment is considered a key factor to enhance the performance of OSCs. The solubility is directly related to the dipole moment. When the value of the dipole moment is high, the solubility in organic solvents also increases and *vice versa*.^28^ In polar solvents, the solubility of compounds increases by increasing the number of polar atoms in the structure of compounds.

**Fig. 9:**
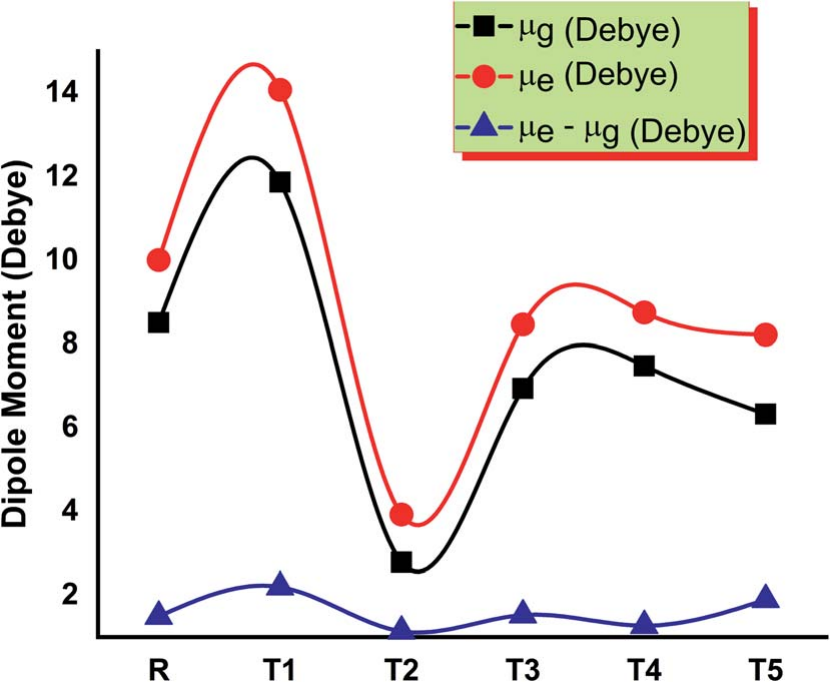
Comparison of the dipole moment of designed molecules T1–T5 and reference R.

The molecule T2 shows the low value of dipole moment in both the gaseous phase and in solvent THF. DSSC's fabrication in the film is somehow affected by dipole moment. Two dipoles are present in such a fashion (anti-parallel), in which they stimulate molecular fabrication, which directly improved the crystallinity. Furthermore, it is said that a higher value of dipole moment increases the charge mobility. The higher value of dipole moment also reduces the disorder between acceptor and donor, resulting in the significantly increased value of charge mobility between acceptor and donor. So, low dipole moment allows low mobility and high stability in the designed molecule.^29^ The dipole moments of T1–T5 molecules and reference R in THF solvent were obtained with functionals B3LYP and TD B3LYP at 6-31G (d,p) DFT method and are given in Table S1.† All molecules have greater dipole moments in THF solvent than the gaseous phase in the order of T1 > T5 > R > T4 > T3 > T2. The order of dipole moment in the gaseous phase is T1 > T5 > R > T4 > T2 > T3. In the gaseous phase, dipole moment values obtained are reference R (8.510861 eV), T1 (11.874998 eV), T2 (5.861444 eV), T3 (6.941926 eV), T4 (7.053962 eV), and T5 (9.05621 eV). The dipole moment values of reference R and compounds T1–T5 are 10.007204, 14.073258, 8.515336, 8.468785, 8.762656, and 10.919255 eV, respectively, given in Fig. 9.

The T2 molecule has a lower dipole moment in gaseous and solvent phases than the four designed (T1, T3, T4, T5) and reference molecule R, confirming their higher stability in organic solar cell devices. The higher values of the dipole moments allow the self-assembly of all designed molecules and the formation of long chains that provide a strong route for the transfer of charge. The dipole moment in excited (*μ*_e_), ground (*μ*_g_), and the difference (*μ*_e_–*μ*_g_) between these two states are given in Table S1,† respectively.

### Open circuit voltage

To calculate the effectiveness of dye sensitized solar cells, open-circuit voltage is the main feature and relates to the competency of the device. The open-circuit voltage of any substance shows the biased junction of current lost from the organic solar cells.^30^ Usually, open circuit voltage initiates from zero and is used to calculate the recombination amount in organic solar cells. The open-circuit voltage can be calculated by eqn (3).^31^3
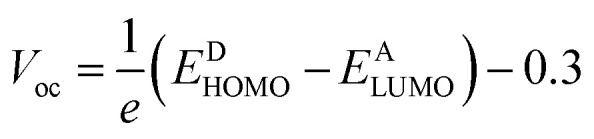


**Fig. 10:**
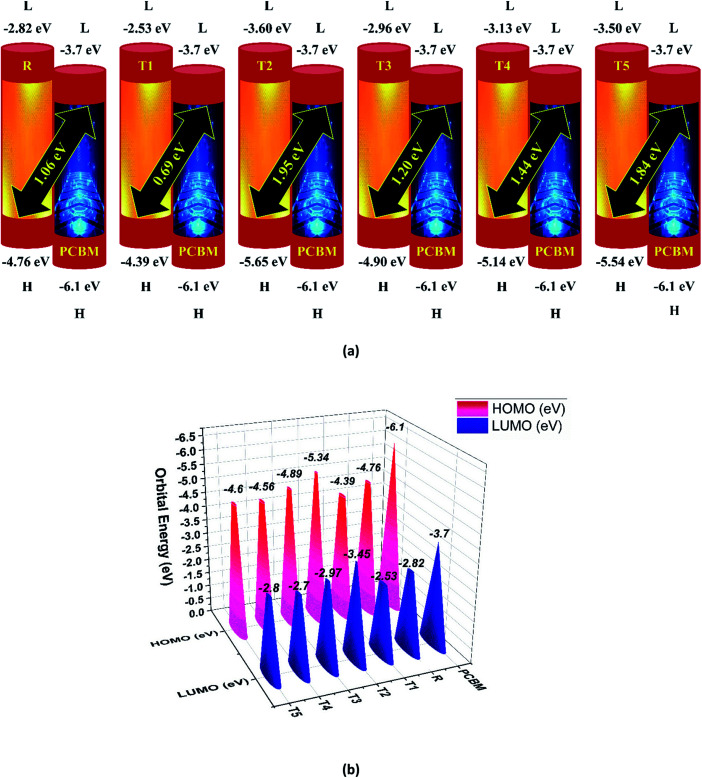
(a) Open circuit voltage (*V*_oc_) of designed donor molecules T1–T5 and reference R with respect to PC71BM acceptor, (b) orbital energy diagram of reference R and designed donor molecules T1–T5 with PC_71_BM acceptor polymer.

Here, *e* is the elementary charge. The value of open-circuit voltage is calculated by the difference between the *E*_LUMO_ of the acceptor and *E*_HOMO_ of the donor of the molecule. Furthermore, this difference in the energy of HOMO and LUMO is directly related to the open-circuit voltage. The designed molecules (T1–T5) are the donors and are suitable to manufacture OPV devices, and due to this reason, we compare our donor molecules with an acceptor molecule named PCBM (6,6-phenyl-C_71_-butyric acid methyl ester).^32^ The open-circuit voltage results obtained from eqn (3) with respect to LUMO_PC_71_BM_–HOMO_donor_ energy gaps are represented in Fig. 10(a). The molecular orbital diagram shows the difference between the *E*_HOMO_ of donor compounds (T1–T5) and *E*_LUMO_ of the PCBM acceptor molecule.

The orbital energy of R and T1–T5 donors with respect to PC_71_BM acceptor are represented in Fig. 10 (b). The LUMO of PC_71_BM is less than the LUMO of donor molecules (R, T1, T2, T3, T4, and T5). This arrangement shifts the charge density from designed donor molecules (T1, T2, T3, T4, T5) to acceptor PC_71_BM, which results in improved photovoltaic properties of all designed molecules.

### Transition density matrix and exciton binding energy

**Fig. 11:**
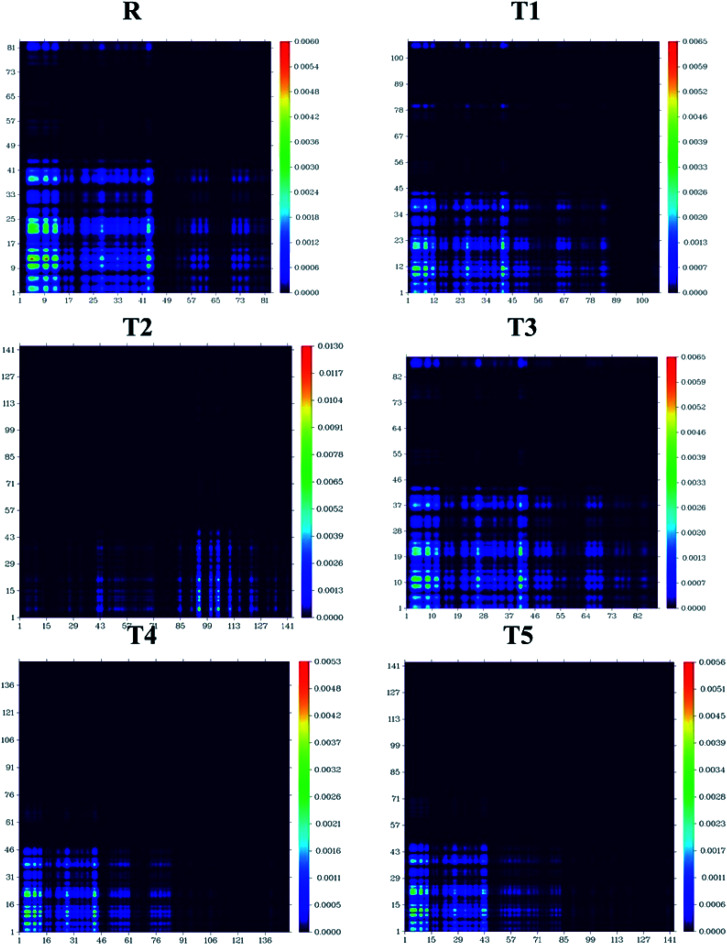
TDMs of designed molecules T1–T5 and reference molecule R in S_1_ state.

The processes of electronic transitions are studied, analyzed, and interpreted by (TDMs) in dye-sensitized solar cells. TDM graphs give the characteristic 3D map between the two eigen states of the newly designed compounds. This 3D map represents associated electron–hole pair distribution and allows them to recognize their lengths of coherence and delocalization.^33^ TDM maps are largely used to explain the excitations of the transfer of charges in solar cells. To examine the emission and absorption up to ten excitation states of reference and other molecules (T1–T5), B3LYP/6-31G (d,p) functional and Multiwfn 3.7 software was used. The effect of hydrogen was neglected due to the negligible contribution of hydrogen atoms. The TDMs results are represented in Fig. 11.

TDM graphs are used to estimate (i) the process of excitations of electrons, (ii) delocalization and localization method of electron–hole pair, and (iii) interaction between the acceptor and donor part of the molecule. We divide our designed compound into acceptor (A) and donor (D) part to evaluate the TDMs. The process of splitting of molecules T1–T5 and reference is shown in Fig. 11. The transition density matrix map shows the coherence of electrons of T1–T5 compounds and reference R having small coherence on acceptor parts and largely on donor moiety of the molecules. The order of interaction coefficients between acceptor and donor parts of molecules is T1 > T3 > R > T5 > T4 > T2. This order of interaction indicates that the coupling of electron and hole of T1 is greater than other compounds.

In an excited state, because of the higher coupling value between the electron and hole (Fig. S2†), the T1 molecule indicates harder and lower exciton dissociation. The values of binding energies show that molecule T1 has the least light-harvesting capability and the least mobility of charges. Thus, the mobility of charges of T1 is lower than all other compounds. The value of charge dissociation and *J*_sc_ (current charge density) for the T2 molecule is higher than others. *E*_b_ (exciton binding energy) is a critical aspect of the rate of separation of charges and the efficiency of dye-sensitized solar cells. The bandgap is the difference of *E*_HOMO_ and *E*_LUMO_ values of molecular orbitals, and the optical gap (*E*_opt_) is 1st excitation energy. The following equation^34^ helps to calculate the binding energies of designed molecules T1–T5 and reference R, theoretically.

4 *E*_b_ = *E*_g_ − *E*_opt_

The analysis of exciton binding energies helps to measure the coulombic force of interaction between holes and electrons. The molecule having a low value of exciton binding energy shows low hole and electron interaction. In the first excited state, the T2 molecule has a higher degree of separation of charges, which corresponds to lower *E*_b_. T2, T4, and T5 molecules have lower *E*_b_ values and correspond to a higher degree of charge separation. The order of exciton binding energy is T2 < T4 < T5 < R < T3 < T1. The detailed comparison between *E*_g_, *E*_opt_ and *E*_b_ is represented in Fig. S3.†

## Conclusion

In this report, five zinc phthalocyanine-based dyes (T1–T5) were designed and investigated for photovoltaic trends using DFT approaches. The UV-vis spectra of T1–T5 molecules and reference R are simulated at the B3LYP/6-31G (d,p) method of DFT. T1 molecule shows greater absorption in gaseous and THF solvent phases than all molecules. In THF solvent, its *λ*_max_ is 751.88 nm. Furthermore, the *E*_g_ value of T1 is lower than T2, T3, T4, and R. The exciton binding energies and transition density matrix (TDM) studies indicate the high charge transfer rate because of the lower *E*_b_ for T1–T5 than R. Open circuit voltage results are achieved with acceptor polymer PC_71_BM. T1 has a lower open-circuit voltage value while T2–T5 molecules have greater open circuit voltage values than reference R. The *V*_oc_ values for T1, T2, T3, T4, and T5 molecules are 0.69, 1.95, 1.20, 1.44, and 1.84 V, respectively. Lower reorganization energy values of T1–T5 show high charge transfer than R. The molecule T1 has lower hole mobility (0.06297 eV) than all other molecules. It is apparent from all discussions that our designed molecules (T1, T2, T3, T4, and T5) can be superb competitors for dye-sensitized solar cells.

## Conflicts of interest

There are no conflicts to declare.

## Supplementary Material

RA-011-D1RA04529F-s001
