# Supplementary material for: Efficient tuning of zinc phthalocyanine-based dyes for dye-sensitized solar cells: a detailed DFT study
Source: RSC Adv. 2021 Aug 12;11(44):27570–82. doi: 10.1039/d1ra04529f (PMC9037920; doi:10.1039/d1ra04529f)
Supplement: RA-011-D1RA04529F-s001 [file RA-011-D1RA04529F-s001.pdf]

Electronic Supplementary Material (ESI) for RSC Advances.
This journal is © The Royal Society of Chemistry 2021

Efficient Tuning of Zinc-phthalocyanine-Based Dyes for Dye-Sensitized Solar Cells-A Detailed DFT Study

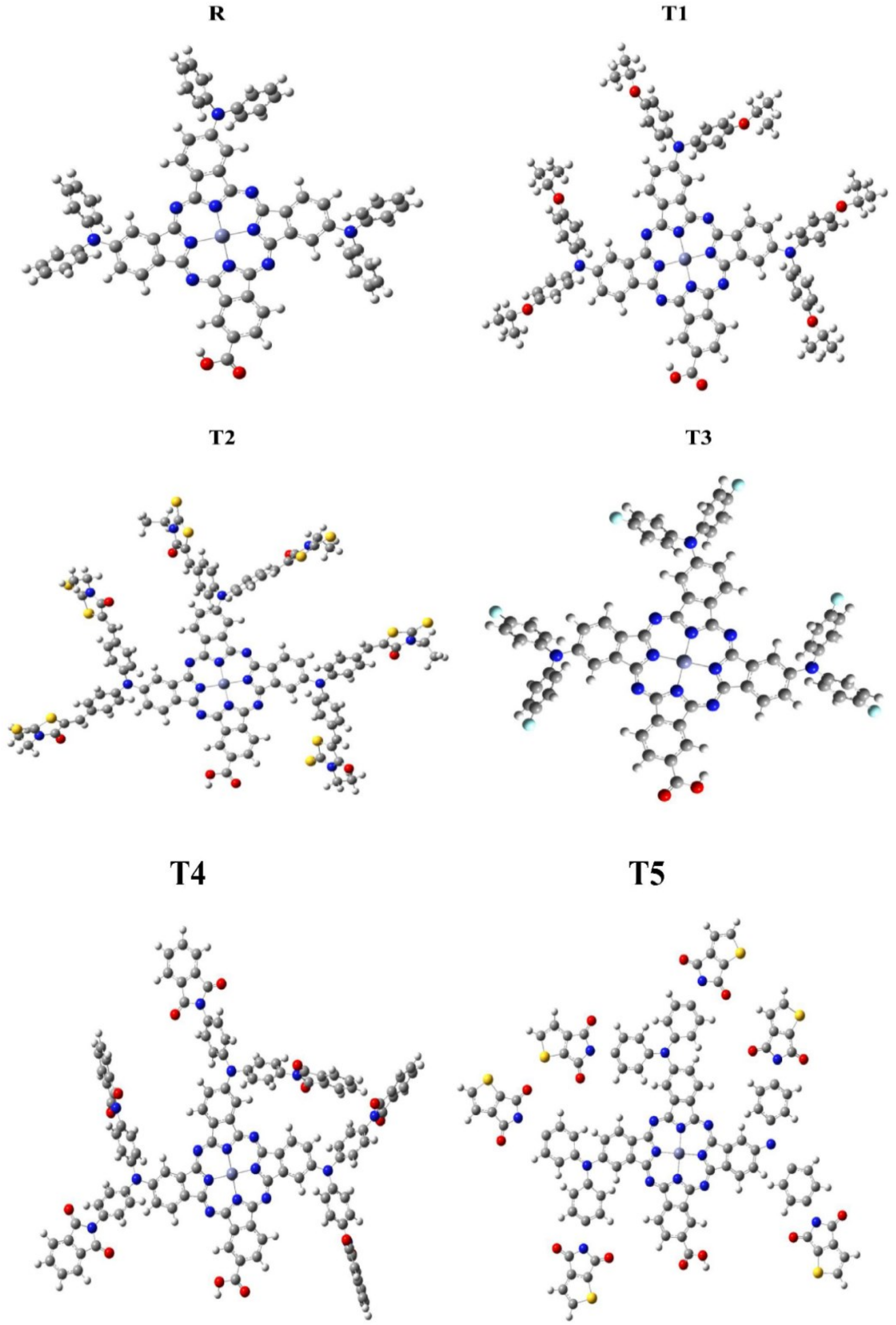

Figure S1. Optimized structures of reference **R** and molecules **T1, T2, T3, T4,** and **T5**.

Efficient Tuning of Zinc-phthalocyanine-Based Dyes for Dye-Sensitized Solar Cells-A Detailed DFT Study

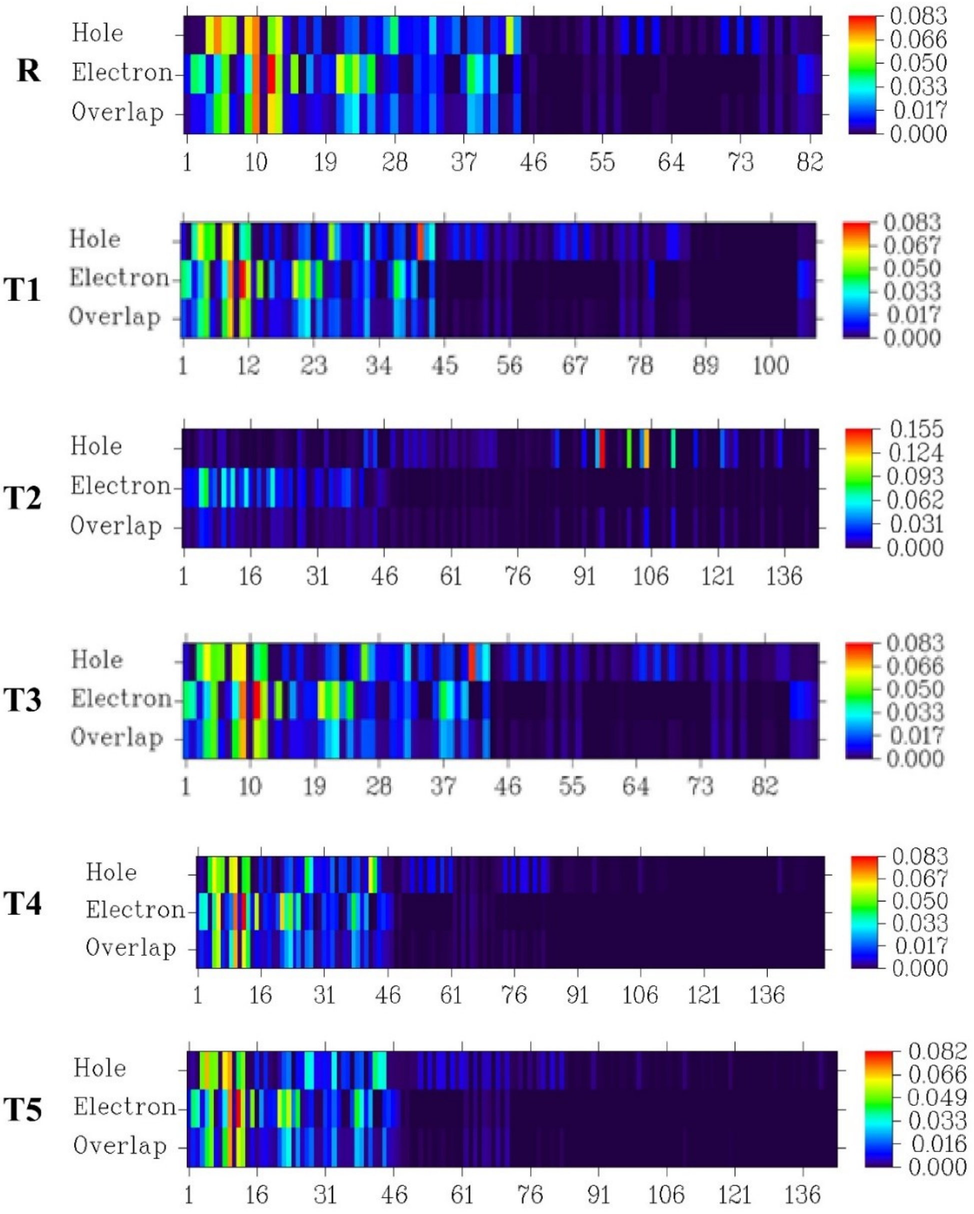

Figure S2. Hole-electron coupling of all designed molecules **T1-T5** and reference **R** as heat map

Efficient Tuning of Zinc-phthalocyanine-Based Dyes for Dye-Sensitized Solar Cells-A Detailed DFT Study

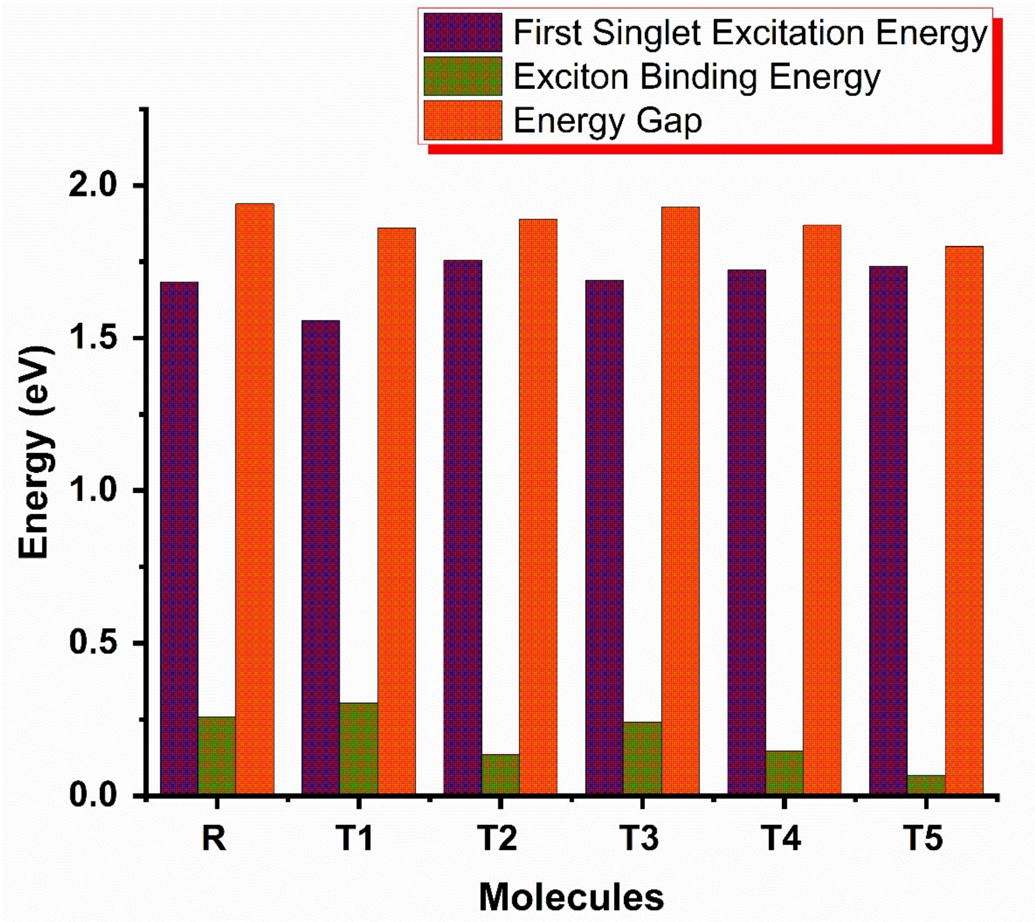

Figure S3. Comparison between E_g, E_{opt} and E_b of all designed molecules **(T1-T5)** and reference **R**.

Table S1: Dipole moment in excited sate μ_e, ground state μ_g, and the difference between these sates for reference **R** and designed molecules **T1, T2, T3, T4** and **T5**

Molecules	μ_g	μ_e	$\mu_e - \mu_g$
R	8.510861	10.007204	1.496343
T1	11.874998	14.073258	2.19826
T2	5.861444	8.515336	2.653892
T3	6.941926	8.468785	1.526859
T4	7.053962	8.762656	1.708694

T5	9.05621	10.919255	1.863045

Table S2: HOMO-LUMO energy gap E_g, first singlet excitation energies E_{opt}, and exciton binding energies E_b

Molecules	$E_g(eV)$	$E_{opt}(eV)$	$E_b(eV)$
R	1.94	1.682	0.257
T1	1.86	1.557	0.303
T2	1.89	1.755	0.215
T3	1.93	1.688	0.258
T4	1.87	1.722	0.248
T5	1.80	1.734	0.066
